# American Academy of Dental Science

**Published:** 1874-01

**Authors:** E. N. Harris


### Proceedings of Societies.

### AMERICAN ACADEMY OF DENTAL SCIENCE.

The sixth annual meeting of the American Academy of Dental Science was held on Monday, September 29, 1873, in Wesleyan Hall, Bromfield street, Boston.

Dr. Daniel Harwood presided.

The forenoon was devoted to a business meeting, at which the various annual reports were read, and new members elected.

Dr. Harwood having served the Academy faithfully and acceptably as its President during the past five years, declined a re-election to that position, and the following gentlemen were elected officers for the ensuing year:

*President.*—Joshua Tucker, M. D.;

*Vice-President.*—Luther D. Shepard, D. D. S.;

*Corresponding Secretary.*—Edward N. Harris, D. D. S.;

*Recording Secretary.*—W. Lewis Tucker, D. M. D.;

*Treasurer.*—George T. Moffatt, M. D., D. M. D.;

*Librarian.*—John Clough, M. D.;

*Board of Censors.*—Elisha G. Tucker, M. D., Jacob L Williams, M. D., Willard W. Codman, M. D.

At two o'clock in the afternoon, the Academy listened to the annual address, prepared by Prof. P. H. Austen, of Baltimore; he having been prevented from attending the meeting by sickness, his address was read by Dr. L. D. Shepard, of Boston. The subject was, "Is Dentistry a Liberal Profession?" and it was treated in the well-known able and scientific manner of the author.

The thanks of the Academy were presented to Prof. Austen, and a copy of the address requested for publication.

Interesting papers were read by Dr. Norman W. Kingsley, of New York, upon "What decided you to become a dentist?" Dr. Asa Hill, of Norwalk, Conn., upon "The progress made in the dental art during the thirty-five years that he had been in practice;" Dr. John T. Codman, of Boston, upon "The best way to spend a vacation."

At five o'clock the members and other invited guests sat down to their sixth anniversary dinner, at the Parker House.

E. N. Harris, D. D. S., *Recording Secretary.*

